# Importance of oral health care in times of COVID-19

**DOI:** 10.31744/einstein_journal/2021CE6706

**Published:** 2021-12-03

**Authors:** Fabiano Vieira Vilhena, Bernardo da Fonseca Orcina, Verônica Caroline Brito Reia, Mariana Ragghianti Zangrando, Rodrigo Cardoso de Oliveira, Paulo Sérgio da Silva Santos

**Affiliations:** 1 TRIALS Bauru SP Brazil TRIALS - Saúde Bucal & Tecnologias, Bauru, SP, Brazil.; 2 Faculdade de Odontologia de Bauru Universidade de São Paulo Bauru SP Brazil Faculdade de Odontologia de Bauru, Universidade de São Paulo, Bauru, SP, Brazil.

Dear Editor,

The infection caused by the severe acute respiratory syndrome coronavirus 2 (SARS-CoV-2) in the host cells occurs by interaction of cleaved viral protein spike with the receptors of angiotensin-converting enzyme 2 (ACE2) and of transmembrane serine protease 2 (TMPRSS2). The presence of ACE2 and TMPRSS2 in the salivary glands make them a reservoir for SARS-CoV-2, leading to cell endocytosis and the viral replication cycle. This knowledge is relevant to better understand and develop strategies to mitigate contamination by droplets.^(1)^

Since the oral cavity is closely related to the progression of the coronavirus 2019 disease (COVID-19), and the oral health status is associated to severity of the condition,^(2)^ adopting preventive measures, such as maintenance of oral health by chemical-mechanical control of oral microbiota, becomes a relevant issue.^(3-6)^

The medical literature has shown the use of broad-spectrum antiseptic mouthwashes demonstrated laboratory results and clinical efficacy in reducing the viral load and symptoms of COVID-19^(3-6)^ (Table 1). The evidence is as recent as the pandemic.

**Table 1:** List of antiseptic mouthwashes used and their respective benefits in treating COVID-19 patients

Antiseptic mouthwash	Benefits
Povidone-iodine (PVP-I)^(3)^	Reducing viral load
Chlorhexidine^(3)^	Reducing viral load
ß-cyclodextrin/Citrox^(4)^	Reducing viral load
Hydrogen peroxide ^(3)^	Reducing viral load
Anionic phthalocyanine derivative (APD) – (Phtalox^®^)^(5,6)^	Reducing viral load, reducing symptoms, reducing severity of disease, reducing length of hospital stay

Therefore, it seems maintenance of oral health, with the correct strategies, does not lead to contraindications or evidence of non-effectiveness in prevention of COVID-19.
