# Supplementary material for: Importance of oral health care in times of COVID-19
Source: Einstein (Sao Paulo). 2021 Dec 3;19:eCE6706. doi: 10.31744/einstein_journal/2021CE6706 (PMC8664280; doi:10.31744/einstein_journal/2021CE6706)
Supplement: Translation

# Translation: Importância dos cuidados com a saúde oral em tempos de COVID-19

**Authors:** Fabiano Vieira Vilhena, Bernardo da Fonseca Orcina, Verônica Caroline Brito Reia, Mariana Ragghianti Zangrando, Rodrigo Cardoso de Oliveira, Paulo Sérgio da Silva Santos

**Affiliations:** 1 Brasil TRIALS - Saúde Bucal & Tecnologias, Bauru, SP, Brasil.; 2 Brasil Faculdade de Odontologia de Bauru, Universidade de São Paulo, Bauru, SP, Brasil.

Caro Editor,

A infecção causada pelo coronavírus 2 da síndrome respiratória aguda grave (SARS-CoV-2) nas células do hospedeiro acontece por meio da interação da proteína viral *spike* clivada com os receptores da enzima conversora da angiotensina 2 (ACE2) e da serina protease 2 transmembrana (TMPRSS2). A presença de ACE2 e TMPRSS2 nas glândulas salivares as torna um reservatório para a SARS-CoV-2, dando início à endocitose celular e ao ciclo de replicação viral. Esse conhecimento é relevante para melhor compreensão e desenvolvimento de estratégias para mitigar a contaminação por meio de gotículas salivares.^(1)^

Sendo a cavidade oral intimamente ligada ao processo evolutivo da doença pelo coronavírus 2019 (COVID-19) e estando a condição de saúde bucal relacionada à gravidade da doença,^(2)^ a adoção de medidas preventivas, como manutenção da saúde oral por meio do controle químico-mecânico da microbiota bucal, passa a ser relevante.^(3-6)^

A literatura médica tem mostrado que o uso de antissépticos bucais de amplo espectro demonstraram resultados laboratoriais e eficácia clínica na redução da carga viral e dos sintomas da COVID-19^(3-6)^ (Tabela 1). As evidências são recentes tanto quanto a pandemia.

**Tabela 1:** Relação dos antissépticos orais utilizados e seus respectivos benefícios no tratamento de pacientes COVID-19

Antisséptico oral	Benefícios
Polivinil pirrolidona iodo (PVP-I)^(3)^	Redução da carga viral
Clorexidina^(3)^	Redução da carga viral
ß-cyclodextrin/Citrox^(4)^	Redução da carga viral
Peróxido de hidrogênio^(3)^	Redução da carga viral
Derivado de ftalocianina (APD) – (Phtalox^®^)^(5,6)^	Redução da carga viral, redução dos sintomas, redução da gravidade da doença, redução do tempo de internação hospitalar

Desse modo, parece que a manutenção da saúde oral, com as estratégias corretas, não leva à contraindicação e nem a evidências de não efetividade na prevenção da COVID-19.
